# VMAT planning study in rectal cancer patients

**DOI:** 10.1186/s13014-014-0219-1

**Published:** 2014-10-16

**Authors:** Jun Shang, Wei Kong, Yan-yang Wang, Zhe Ding, Gang Yan, Hong Zhe

**Affiliations:** Department of Radiation Oncology, General Hospital of Ningxia Medical University, No.804 Shengli Str, Yinchuan, 750004 Ningxia China

## Abstract

**Background:**

To compare the dosimetric differences among fixed field intensity-modulated radiation therapy (IMRT), single-arc volumetric-modulated arc therapy (SA-VMAT) and double-arc volumetric-modulated arc therapy (DA-VMAT) plans in rectal cancer.

**Method:**

Fifteen patients with rectal cancer previously treated with IMRT in our institution were selected for this study. For each patient, three plans were generated with the planning CT scan: one using a fixed beam IMRT, and two plans using the VMAT technique: SA-VMAT and DA-VMAT. Dose prescription to the PTV was 50 Gy in 2 Gy per fraction. Dose volume histograms (DVH) for the target volume and the organs at risk (small bowel, bladder, femoral heads and healthy tissue) were compared for these different techniques. Monitor units (MU) and delivery treatment time were also reported.

**Results:**

DA-VMAT achieved the highest minimum planning target volume (PTV) dose and the lowest maximal dose, resulting in the most homogeneous PTV dose distribution. DA-VMAT also yielded the best CI, although the difference was not statistically significant. Between SA-VMAT and IMRT, the target dose coverage was largely comparable; however, SA-VMAT was able to achieve a better V95 and V107. VMAT showed to be inferior to IMRT in terms of organ at risk sparing, especially for the small bowel. Compared with IMRT, DA-VMAT increased the V15 of small bowel nearly 55 cc. The MU and treatment delivery time were significantly reduced by the use of VMAT techniques.

**Conclusion:**

VMAT is a new radiation technique that combines the ability to achieve highly conformal dose distributions with highly efficient treatment delivery. Considering the inferior role of normal tissue sparing, especially for small bowel, VMAT need further investigation in rectal cancer treatment.

## Background

Rectal cancer radiotherapy is a complex problem because of the shape of target volumes and the need of minimizing the involvement of organs at risk (OAR) such as small bowel, bladder and femur heads [1]. Lots of planning studies have demonstrated the advantages of Intensity-modulated radiation therapy (IMRT) in target coverage and normal tissue sparing over three-dimensional conformal radiotherapy (3D-CRT) for rectal cancer patients [2-4]. However, drawbacks of the IMRT technique have also been reported. The prolonged delivery time per fraction may worsen the accuracy of treatment because of increased intra-fractional patient motion. In addition, more monitor units (MU) and a bigger volume of normal tissue exposed to lower radiation dose would increase the possibility of radiation-induced secondary malignancies [5,6].

Volumetric-modulated arc therapy (VMAT) is a technique enabling an intensity-modulated dose to be delivered during a continuous gantry rotation. For the dynamically moving multileaf collimator (MLC), variable dose rate and gantry rotation speed during the rotation, VMAT could achieve highly conformal dose distributions and is essentially an alternative form of IMRT [7,8]. Moreover, the improvement in treatment delivery efficiency and the reduction in MU usage of this novel technique could overcome the reported shortages of IMRT [9].

Most of the planning studies in various tumor sites have compared VMAT with either fixed field IMRT or 3D-CRT techniques [10-13]. However, the efficacy of VMAT may be organ-site dependent. In rectal cancer, VMAT has clear superiority over 3D-CRT with regard to improving dose conformity and OAR sparing [14,15]. However, the distinction between VMAT and fixed field IMRT is not well documented. To our knowledge, there is only one planning study comparing IMRT and VMAT in rectal cancer until now. This study was done by Cilla et al. [16] in Italy. However, the difference of single-arc (SA)-VMAT and double-arc (DA)-VMAT for rectal cancer patients was not compared in Cilla’s study. In present study, we compared the dosimetric parameters among fixed field IMRT, SA-VMAT and DA-VMAT for rectal cancer patients and evaluated the efficacy of VMAT technique in rectal cancer treatment.

## Methods

### Patient and simulation

Fifteen patients with pathologically proven rectal cancer in locally advanced stage, subjected to radical postoperative radiotherapy were selected for this study. There were nine males and six females. The median age of these patients was 59 (range, 38–79). The research protocol was reviewed and approved by Ethics Committee of General Hospital of Ningxia Medical University. All patients were simulated in prone position with full bladder and immobilized with Belly-board to dislocate anteriorly as much as possible intestinal loops of small bowel. Computed tomography (CT) scans were acquired with 3-mm slice thickness through the L1 vertebral body to 5 cm below the perineum.

### Target volume definition

Target volumes were outlined on the planning CT scan by the treating radiation oncologist. The clinical target volume (CTV) was delineated according to published consensus guidelines [17]. The planning target volume (PTV) was defined with margins around the CTV of: 0.5 cm lateral, 1 cm superior-inferior and 0.8 cm anterior-posterior. Bladder, small bowel and femur heads were contoured as OAR. The small bowel loops was outlined 3 cm above and below the PTV, and the bladder and femur heads were fully outlined. In addition, the healthy tissue was defined as the patient’s volume included in the CT dataset minus the PTV volume.

### Dose constraints for PTV and normal tissue

Dose prescription to the PTV was 50 Gy in 2 Gy per fraction. Dose constraints for the PTV were as follows; (1) ≥ 98% of the PTV receives ≥ 93% of the prescribed dose, (2) ≤ 10% of the PTV receives ≥ 105% of the prescribed dose, (3) ≤ 5% of the PTV receives ≥ 110% of the prescribed dose. (4) None of the PTV receives ≥ 115% of the prescribed dose. For OAR, small bowel V35 Gy < 180 cc, V40 Gy < 100 cc, V45 Gy < 65 cc and no small bowel volume should receive 50 Gy; bladder D40% < 40 Gy, D15% < 45 Gy and no bladder volume should receive 50 Gy; femur heads D40% < 40 Gy, D25% < 45 Gy and no femur heads should receive 50 Gy.

### Planning techniques

Three sets of plans, IMRT, SA-VMAT, and DA-VMAT, were created on the Eclipse Treatment Planning System (Version 11.0; Varian Medical Systems), and calculated using the Anisotropic Analytical Algorithm, using a 2.5 mm calculation grid, a tissue heterogeneity correction was applied. The same dose constraint parameters of PTV and normal tissue were used for IMRT and VMAT planning.

IMRT planning: IMRT plans were optimised with Direct Machine Parameter Optimization (DMPO) approach using seven coplanar beams (0, 50,100,150, 210, 260 and 310) with a dose rate of 400 MU/min and beam energy of 6-MV photons. The maximal number of segments was set to 100 with a minimal number of MU per segment equal to 3.

VMAT planning: VMAT plans were calculated using 6-MV photons with a maximum variable dose rate of 600 MU/min. Single-arc corresponded to a single 360° rotation and double-arc to two coplanars arcs of 360° sharing the same isocenter and optimised independently. These two arcs were delivered with opposite rotation (clock and counter-clock) and so minimize the off-treatment between the two beams time about 25 seconds. For SA-VMAT, field size and collimator rotation were determined by the automatic tool from Eclipse to encompass the PTV. We controlled that the collimator was always rotated to a value different from zero in order to avoid tongue and groove effect. For DA-VMAT, the first arc was similar to the one defined in the single-arc process except for the rotation of the collimator, which was 360 minus X for the second arc (X corresponded to the rotation of the collimator of the first arc).

### Plan evaluation and comparison

Dosimetric parameters to analyze target coverage and dose distribution in the PTV are as follows; (1) mean dose, (2) Vn Gy, percentage of the volume receiving radiation ≥ n Gy, (3) D98%, minimum dose to 98% of the PTV, (4) D2%, maximum dose to 2% of the PTV, (5) conformality index (CI) defined as the volume encompassed by the 95% isodose divided by the PTV volume. For OAR, the analysis included the mean dose, the maximum dose expressed as D2% and a set of appropriate Vn Gy and Dn values. For healthy tissue, we detailed the volume of the body minus PTV receiving low doses (V5, V10, and V20 Gy). The number of MU per fraction required for each plan and the treatment delivery time (from beam-on to beam-off) was reported.

### Statistical analysis

To appraise the difference between the techniques, Wilcoxon non-parametric two-sample test was applied. Data were considered statistically significant at *p* < 0.05.

## Results

### PTV volumes, target coverage, conformity, and dose homogeneity

**Figure 1:**
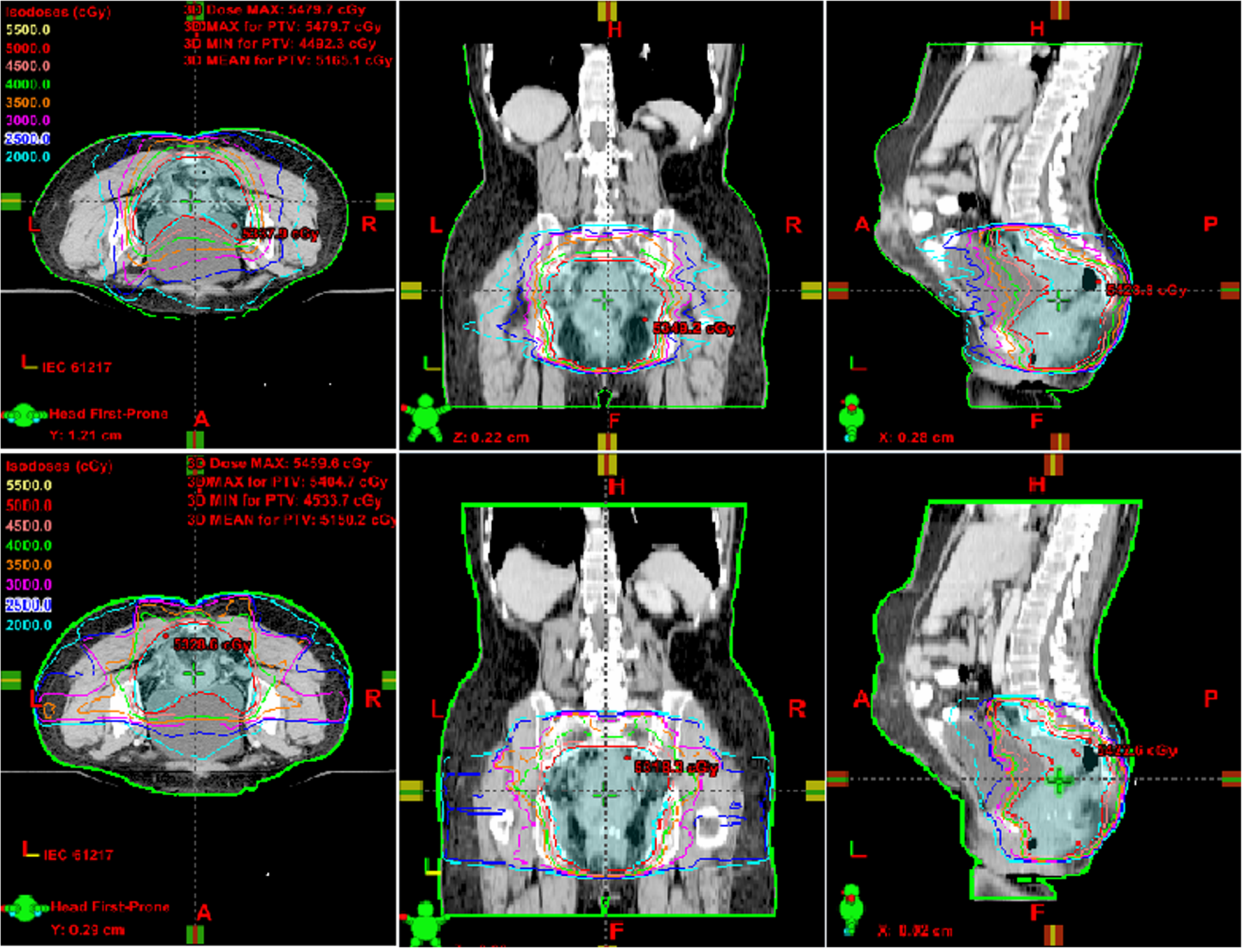
**Representation of isodose distribution in axial, coronal and sagittal views for fixed IMRT (the first line) and DA-VMAT (the second line).** IMRT: intensity modulated radiotherapy, DA-VMAT: double-arc volumetric modulated arc therapy.

**Figure 2:**
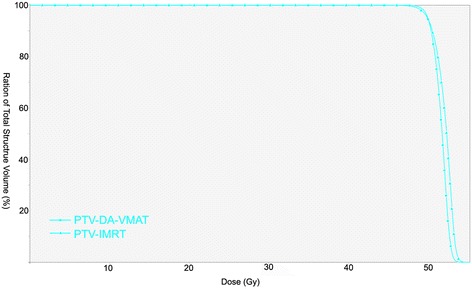
**Dose-volume histograms for PTV of an individual patient in present study.** IMRT: intensity modulated radiotherapy, DA-VMAT: double-arc volumetric modulated arc therapy.

The median of the PTV volume outlined in the 15 patients was 1317.2 cc (range, 1048.4–1587.3). The IMRT, SA-VMAT and DA-VMAT plan met the prescription requirements for the PTV in all cases. Dosimetric parameters for the PTV in these three plans were listed and compared in Table 1. DA-VMAT achieved the highest minimum PTV dose and the lowest maximal dose, resulting in the most homogeneous PTV dose distribution. DA-VMAT also yielded the best CI, although the difference was not statistically significant. Between SA-VMAT and IMRT, the target dose coverage was largely comparable; however, SA-VMAT was able to achieve a better V95 and V107. Dose distribution and DVH of PTV for a typical patient are shown in Figures 1 and 2.

**Table 1:** **Dosimetric parameters comparison among IMRT, SA-VMAT and DA-VMAT technique for PTV (mean ± standard deviation)**

	**IMRT**	**SA-VMAT**	**DA-VMAT**	***P*** **(IMRT vs SA-VMAT)**	***P*** **(IMRT vs DA-VMAT)**
Dmean (Gy)	52.0 ± 0.2	52.0 ± 0.4	51.6 ± 0.2	0.167	0.001
D2% (Gy)	54.1 ± 0.2	54.0 ± 0.8	53.1 ± 0.3	0.366	0.001
D98% (Gy)	49.2 ± 0.3	49.3 ± 0.2	49.5 ± 0.2	0.230	0.015
V95 (%)	99.6 ± 0.3	99.9 ± 0.1	99.9 ± 0.1	0.001	0.001
V107 (%)	10.2 ± 5.1	5.3 ± 3.9	0.6 ± 0.4	0.017	0.001
CI	1.23 ± 0.05	1.28 ± 0.07	1.21 ± 0.07	0.006	0.305
MU	499.0 ± 41.6	418.5 ± 41.9	438.8 ± 31.7	0.001	0.001
Treatment time (min)	8.0 ± 0.7	1.5 ± 0.2	3.0 ± 0.3	0.001	0.001

IMRT: intensity modulated radiotherapy, SA-VMAT: single-arc volumetric modulated arc therapy, DA-VMAT: double-arc volumetric modulated arc therapy, PTV: Planning Target Volume, CI: conformity index, MU: monitor units.

### OAR

**Figure 3:**
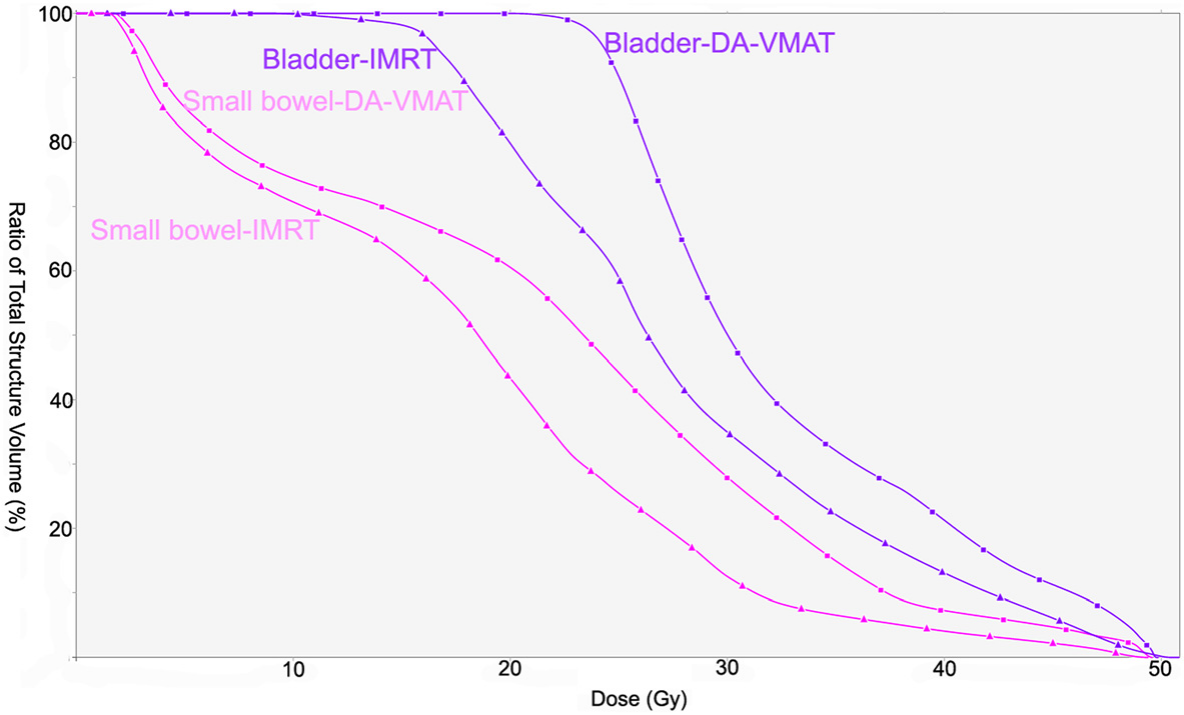
**Dose-volume histograms for small bowel and bladder of an individual patient in present study.** IMRT: intensity modulated radiotherapy, DA-VMAT: double-arc volumetric modulated arc therapy.

**Figure 4:**
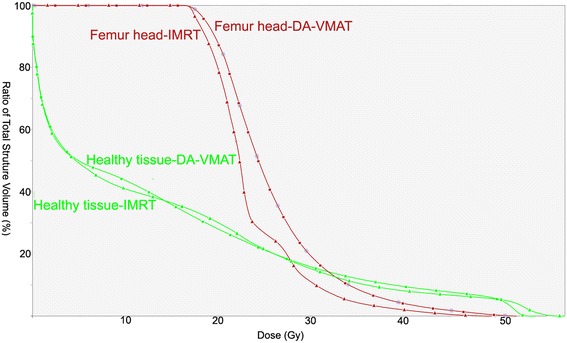
**Dose-volume histograms for femur head and healthy tissue of an individual patient in present study.** IMRT: intensity modulated radiotherapy, DA-VMAT: double-arc volumetric modulated arc therapy.

Table 2 shows the dosimetric parameters of the OAR including the small bowel, bladder, femur heads and healthy tissue. For small bowels, no sparing effort was devoted to this organ. The DVH parameters, Dmean, D2%, V15 and V30, were increased when use VMAT technique. For bladder, planning objectives were met by all techniques and no relevant difference was observed between DA-VMAT and IMRT, except the maximal dose. In addition, SA-VMAT increased the Dmean, V40 and V50 of bladder, when compared with IMRT. Concerning femurs heads, SA-VMAT and DA-VMAT both showed a significant increase in mean dose and D10. For healthy tissue, although V20 was significantly lower for VMAT than for IMRT, V5 and V10 were found to be significantly larger for VMAT with respect to IMRT. DVH of normal tissue for a typical patient are shown in Figures 3 and 4.

**Table 2:** **Dosimetric parameters comparison for OAR (mean ± standard deviation)**

		**IMRT**	**SA-VMAT**	**DA-VMAT**	***P*** **(IMRT vs SA-VMAT)**	***P*** **(IMRT vs DA-VMAT)**
Small bowel	Dmean (Gy)	12.1 ± 8.0	15.5 ± 7.7	15.3 ± 7.6	0.001	0.002
	D2% (Gy)	35.7 ± 12.1	40.0 ± 10.2	39.6 ± 10.0	0.005	0.009
	V15 (cc)	132.0 ± 110.2	185.8 ± 130.0	186.4 ± 128.8	0.004	0.005
	V30 (cc)	48.8 ± 20.0	77.5 ± 21.0	78.8 ± 21.5	0.001	0.002
Bladder	Dmean (Gy)	39.0 ± 3.3	42.2 ± 4.0	40.0 ± 3.6	0.001	0.074
	D2% (Gy)	52.9 ± 0.6	53.2 ± 0.7	52.4 ± 0.5	0.132	0.005
	V30 (cc)	437.1 ± 156.5	479.4 ± 176.5	463.2 ± 191.2	0.061	0.460
	V40 (cc)	281.9 ± 118.9	366.2 ± 155.8	321.1 ± 167.1	0.002	0.140
	V50 (cc)	112.0 ± 55.6	185.7 ± 107.4	151.1 ± 115.6	0.001	0.112
Femur heads	Dmean (Gy)	19.9 ± 4.1	23.2 ± 3.4	24.3 ± 2.8	0.025	0.012
	D10% (Gy)	28.2 ± 5.5	29.6 ± 4.3	31.5 ± 4.1	0.173	0.047
Healthy tissues	V5 (cc)	9454.9 ± 1999.3	9827.4 ± 2153.3	9815.3 ± 2142.0	0.001	0.001
	V10 (cc)	7833.9 ± 1591.8	8167.6 ± 1789.4	8331.8 ± 1733.7	0.013	0.001
	V20 (cc)	5224.2 ± 1084.5	4721.8 ± 884.3	4750.7 ± 876.5	0.002	0.005

OAR: organ at risk, IMRT: intensity modulated radiotherapy, SA-VMAT: single-arc volumetric modulated arc therapy, DA-VMAT: double-arc volumetric modulated arc therapy.

### MU and treatment delivery time

The MU was significantly reduced by the use of VMAT. The lower MU combining with less beam mode-up procedures resulted in a much shorter treatment time with VMAT. Compared to a delivery in 8 min for IMRT, treatment delivery time with VMAT was definitely shorter and was 1.5 and 3.0 minutes for single and double arcs, respectively (Table 1).

## Discussion

Arc therapy was initially reported in rectal cancer by Duthoy et al. [14] in a planning study comparing 3D-CRT and Intensity-modulated arc therapy (IMAT). They found IMAT plans were deliverable within a 5–10-minute time slot, and resulted in a lower dose to the small bowel than 3D-CRT plans, without creating significant underdosages in the PTV. Richetti et al. [15] reported on their technical and clinical experience of 25 patients with locally advanced rectal cancer treated with VMAT and performed a planning comparison with a matched cohort of patients who underwent conventional conformal radiotherapy. VMAT improved conformality of doses, presented similar target coverage with lower maximum doses, significant sparing of femur heads and significant reduction of integral and mean dose to healthy tissue. Acute toxicity was limited to Grade 1–2 diarrhea in 40% and Grade 3 in 8% of VMAT patients, 45% and 5% of conventional conformal radiotherapy patients, compatible with known effects of concomitant chemotherapy. To our knowledge, there is only one planning study comparing IMRT and VMAT in rectal cancer until now. This study was done by Cilla et al. [16] in Italy. VMAT had the highest level of conformality, but the dose distribution across PTV was less homogenous than with IMRT and 3D-CRT were found in their study. With respect to the V15 objective, they found small bowel irradiation to be significantly reduced to 171.2 cc with VMAT, compared with 199.5 cc and 227.4 cc with IMRT and 3D-CRT, respectively.

In present study, we evaluated the feasibility and efficiency of IMRT, SA-VMAT and DA-VMAT for the treatment of rectal cancer. The major advantage of using the VMAT technique is the significant reduction in treatment times with its potential advantages in lower MU. This improvement is mainly from the elimination of all the non-beam-on times, such as MLC movements to realize the various segments of IMRT beams or gantry motion to reach the fixed position. The reduction of treatment delivery time is clinically relevant considering the patient comfort and infra-fraction motion. The higher delivery efficiency also allowed for more time for image-guided radiotherapy to further reduce the treatment margin and toxicity [18].

Although there is a clear advantage of VMAT in terms of faster delivery and lower MU, this needs to be balanced with the dosimetric differences. Compared with IMRT, DA-VMAT provided better coverage of target but not the normal tissue sparing, especially for the small bowel, which was different from the conclusion made in Cilla’s study. The first reason of the difference is that the healthy tissue which received dose below 20 Gy was increased when using VMAT technique in present analysis. Although this finding was supported by other dosimetric studies [19-21], the low dose bath may enlarge the V15 of small bowel. Another reason of the different small bowel sparing effect is that we enrolled postoperative rectal cancer patients in present study. It was shown that patients who receive postoperative radiotherapy have a larger portion of small bowel in the pelvis [22]. Lastly, we followed the dose–volume constraints of RTOG0822 protocol and did not make lower dose-volume constraint in the planning procedure, which may contribute to the enlargement of V15 for small bowel.

## Conclusion

VMAT is a new radiation technique that combines the ability to achieve highly conformal dose distributions with highly efficient treatment delivery. Considering the inferior role of normal tissue sparing, especially for small bowel, VMAT need further investigation in rectal cancer treatment.
